# Performance Comparison of Tungsten-Halogen Light and Phosphor-Converted NIR LED in Soluble Solid Content Estimation of Apple

**DOI:** 10.3390/s23041961

**Published:** 2023-02-09

**Authors:** Hoyoung Lee, Sungho Cho, Jongguk Lim, Ahyeong Lee, Giyoung Kim, Doo-Jin Song, Seung-Woo Chun, Min-Jee Kim, Changyeun Mo

**Affiliations:** 1 Department of Mechatronics Engineering, Korea Polytechnics, 56 Munemi-ro 448 beon-gil, Bupyeong-gu, Incheon 21417, Republic of Korea; 2 Department of Smart Automation, Korea Polytechnics, 398 Sujeong-ro, Sujeong-gu, Seongnam-si 13122, Republic of Korea; 3 Rural Development Administration, 310 Nongsaengmyeng-ro, Deokjin-gu, Jeonju 54875, Republic of Korea; 4 Interdisciplinary of Program in Smart Agriculture, Kangwon National University, 1 KNU Ave., Chuncheon 24341, Republic of Korea; 5 Agriculture and Life Sciences Research Institute, Kangwon National University, 1 KNU Ave., Chuncheon 24341, Republic of Korea; 6 Department of Biosystems Engineering, College of Agriculture and Life Science, Kangwon National University, 1 KNU Ave., Chuncheon 24341, Republic of Korea

**Keywords:** pc-NIR LED, NIR light source, miniature spectrometer system, Vis-NIR spectroscopy, soluble solid content (SSC)

## Abstract

A Tungsten-Halogen (TH) lamp is the most popular light source in NIR spectroscopy and hyperspectral imaging, which requires a warm-up to reach very high temperatures of up to 250 °C and take a long time for radiation stabilization. Consequently, it has a large enough volume to enable heat dissipation to prevent the thermal runaway of the electric circuit and turn out its power efficiency very low. These are major barriers for miniaturizing spectral systems and hyperspectral imaging devices. However, TH lamps can be replaced by pc-NIR LEDs in order to avoid high temperature and large volume. We compared the spectral emission of the available commercial pc-NIR LEDs under the same condition. As a replacement for the TH lamp, the VIS + NIR LED module was developed to combine a warm-white LED and pc-NIR LEDs. In order to feature out the availability of the VIS + NIR LED module against the TH lamp, they were used as the light source for evaluating the Soluble Solid Content (SSC) of an apple through VIS-NIR spectroscopy. The results show a remarkable feasibility in the performance of the partial least square (PLS) model using the VIS + NIR LED module; during PLS calibration, the correlation coefficient (R) values are 0.664 and 0.701, and the Mean Square Error (MSE) values are 0.681 and 0.602 for the TH lamp and VIS + NIR LED module, respectively. In VIS-NIR spectroscopy, this study indicates that the TH lamp could be replaceable with a warm-white LED and pc-NIR LEDs.

## 1. Introduction

In spectroscopy and hyperspectral imaging, the visible (Vis) and near infrared (NIR) wavebands have been frequently used for medical and physiological diagnostics and research. Specifically, the 400–1000 nm region is more effective for food safety inspection, including the rapid evaluation of the internal quality of agricultural products, such as freshness, ripeness, and soluble solid content (SSC). Through VIS-NIR hyperspectral imaging [1,2], detection of the melamine portion in milk powder and the early detection of the chilling injury of fresh bell pepper is possible. Using NIR spectroscopy, the internal quality of wine grapes is monitored during on-vine ripening and harvesting [3]. 

Moreover, this waveband is coincident with the spectral response domain of Charge-Coupled Device (CCD) and Complementary Metal-Oxide Semiconductor (CMOS) sensors, which are the most common silicon-based optical sensors for measuring spectral intensities. With the advancement in semiconductor technology, optical sensors become more compact and reliable; consequently, spectral and imaging devices can be miniaturized. A spectrometer with a small form factor, which supports wireless communication, has been introduced [4]. A handheld spectrometer has been used for recycling polymer commodities [5]. A miniature spectral imaging system that provides high spectral and spatial resolution using a sensor array and single variable Liquid Crystal (LC) retarder has been presented [6]. Compact hyperspectral cameras have been proposed for combining the spectral and spatial resolutions, with the convenience of a snapshot camera [7].

However, the light source, which is the remaining part, is an obstacle for system miniaturization. In hyperspectral imaging or spectral system, the tungsten-halogen (TH) lamp is one of practical approaches for providing a full spectrum between the Vis and NIR wavelengths; in order to estimate the nitrogen concentration in soil, two strip TH-lamps have been employed as the lighting source of a hyperspectral imaging system [8], the capsaicinoid content of red pepper powder has been determined by measuring the reflectance of the radiation from a TH lamp [9], and the spectral detection of campylobacter colonies has been accomplished [10]. In principle, the radiation from the TH lamp is driven by the chemical reaction of the halogen cycle, which requires a very high temperature of up to 250 °C [11]. Such high temperatures reduce the lifetime of the TH lamp, and all the components are voluminous in order to improve heat dissipation. In addition, it is highly sensitive in emitting radiant flux at the internal temperature and requires a considerable warmup time for the spectral radiation being stabilized. Thus, its light production efficiency is low and miniaturization of the light source is difficult [12].

With recent advances of light emitting diode (LED) technology, bulb-type light sources, including incandescent light bulbs, have been replaced by LEDs. The first one was the infrared constructed from a gallium arsenide (GaAs) substrate in 1961 [13], which triggered the development of LEDs with emission wavebands down to green; from the infrared to red, and gradually, to orange-red, orange, yellow and green. The remaining blue was developed in 1994 [14]. Therefore, the white-colored can be manufactured by placing red, green, and blue diodes on the same silicon chip. The first white LED was not explicitly white; the red, green, and blue wavebands were blended to cover the white waveband, and different colored lights could be generated by determining the suitable proportions [15]. In addition, the white-colored can be manufactured using phosphor, which emits homogeneous white light when excited by ultraviolet or violet radiation. This type of white LED comprises ultraviolet or violet-colored diodes covered by a phosphor-coated layer [16]. The phosphor-conversion LED has been further developed to generate white light with increased power efficiency, brightness, and compactness, and has become the most popular light source due to higher luminous efficiency.

Phosphor-converted near-infrared (pc-NIR) LEDs were recently introduced in the market for broadband NIR radiation [17,18]. Because it can overcome bulkiness, a short lifetime, temperature-dependent instability, and high operation temperature, a pc-NIR LED appear to be a promising alternative against TH lamp [19]. More efficient near-infrared (NIR) phosphor has been proposed to provide a smarter and more sensitive light source for night vision [20]. The high photoelectric efficiency of pc-NIR LEDs can provide a compact NIR light source with high spectral emission and thermal stability [21]. Thus, with the help of pc-NIR LED, it is possible to take a revolutionary step to bring out entirely new, and previously unattainable applications of NIR spectroscopy, and the appearance of the autonomous and portable NIR spectrometer, which can do on-site spectral measurements in agri-food or natural medicine industry [22].

The aim of this study is to compare the performances of the tungsten-halogen light and the available commercial pc-NIR LEDs for radiation from 650 to 1000 nm and develop a VIS + NIR LED module to emit radiation from 400 to 1000 nm. To determine the possibility of replacing the TH lamp by the VIS + NIR LED module, spectral analysis is performed for estimating the SSC of a Fuji apple based on the transmittance through it where the developed VIS + NIR LED module and TH lamp are used as the light source, respectively. In order to evaluate the SSC of a Fuji apple, PLSR (Partial Least Squares Regression) model was used, which is the most popular in the Vis/NIR spectroscopy [23,24,25].

## 2. Materials and Methods

The radiation spectra were compared for evaluation of the spectral intensity and characteristics of the existing commercialized pc-NIR LEDs. For an appropriate comparison, they were measured under the same condition in electrical, thermal, and optical properties; the metal core printed circuit board (MCPCB) was designed and manufactured for each LED, and the electric power was supplied as similarly as possible. Then, using a combination of a kind of pc-NIR LED and a warm white LED, the lighting system was developed to obtain a spectral intensity like that through a 20 W TH lamp. Regarding predicting the SSC of the apple, the TH lamp and the developed LED module were featured out of the performance through PLS regression.

### 2.1. Comparison of the Spectral Intensity of the pc-NIR LED Modules

**Figure 1:**
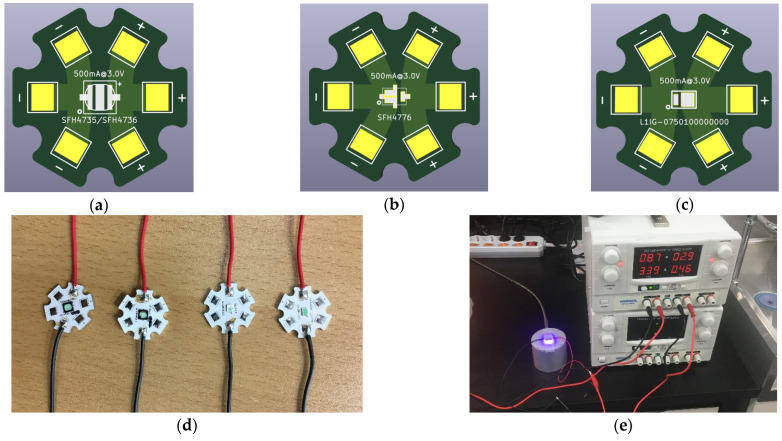
Schematic of (**a**) SFH4735 and SFH4736, (**b**) SFH4776, and (**c**) L1IG-0750100000000; (**d**) SFH4735, SFH4736, SFH4776, and L1IG-0750100000000 LED modules with MCPCBs; (**e**) Vis/NIR spectrum data acquisition system with a power supply, irradiation integration sphere, and spectrometer.

For appropriate comparison of the spectral intensities of four kinds of pc-NIR LEDs, which are SFH4735, SFH4736, SFH4776 (OSRAM GmbH, Munich, Germany), and L1IG-0750100000000 (LUMILEDS, CA, USA), MCPCBs were designed and manufactured to provide the same condition of thermal and electrical properties. As shown in Figure 1a–c, all these LEDs have the same base composed of aluminum, which has a relatively higher thermal conductivity than a nonmetallic base. In terms of the current-carrying capacity of the LED silicon, a wide trace width on the MCPCB provides low electric resistance, which is beneficial for delivering a high current.

Hence, the developed MCPCB is optimal for providing high thermal conductivity, low electrical resistance, and the stable fixture for optical layout for the pc-NIR LEDs, as depicted in Figure 1d. Under the same conditions, the spectral intensities of the four kinds of pc-NIR LEDs were acquired using a VIS/NIR spectrometer (HR4PRO-VIS-NIR-ES, Ocean Optics, Orlando, FL, USA) and irradiance integrating sphere (AvaSphere-50, Avantes, Apeldoorn, The Netherlands), as shown in Figure 1e. With the benchtop power supply, the forward voltage (V_f_) and forward current (I_f_) of each pc-NIR LED was constantly provided, and the total electrical power was maintained as similarly as possible among them.

**Figure 2:**
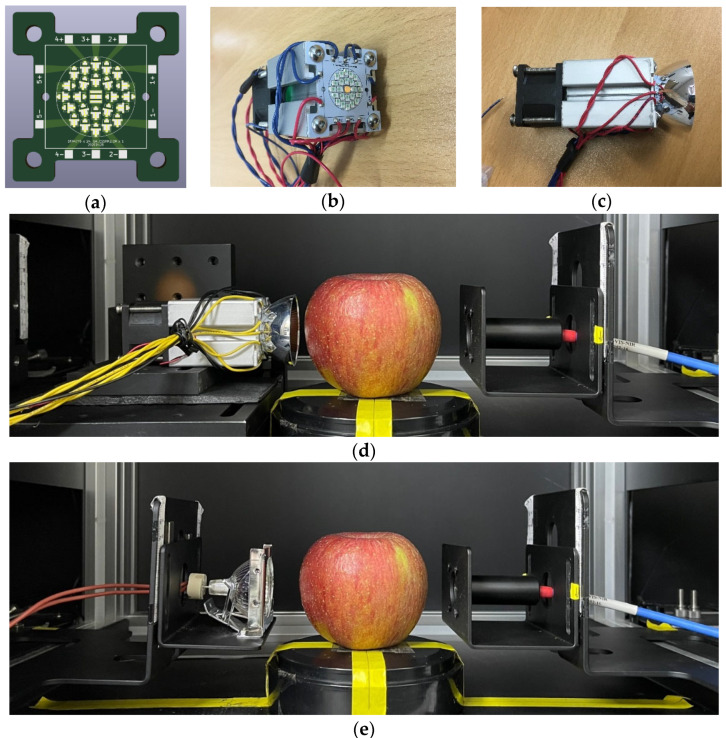
(**a**) MCPCB design for 24 pc-NIR LEDs and a warm white LED; (**b**) LED module assembly, AL extrusion, and blower fan; (**c**) Reflector attachment for concentrating the Vis/NIR radiation; (**d**,**e**) spectral data acquisition apparatus for the SSC estimation of apple using the LED module and TH lamp.

The spectral data were compared to examine the spectral response at a wavelength range of 200 to 1050 nm. The spectral response of a 12-V/20-W TH lamp (MR16, OSRAM, Germany), a pc-NIR LED was chosen to provide wider and higher radiation at a wavelength range of 650 to 1000 nm, and then used to make the LED matrix, which is able to provide the emission in the visible and NIR wavelength ranges, similar to that of a TH lamp as shown in Figure 2d,e.

### 2.2. Comparison of the SSC Evaluation for the LED Module and TH Lamp

Electromagnetic radiation at a wavelength range of 400–1000 nm is often used to quantify the SSC or acidity for the nondestructive evaluation of agricultural products. The TH lamp is extensively used in hyperspectral imaging systems or NIR spectroscopic systems as the light source for a broad band of wavelength. Even though the pc-NIR LED is one of the light sources that could replace the TH lamp, its radiation intensity has a lack of 500–700 nm in waveband. In order to fully provide 400–1000 nm radiation as close as possible to that of a TH lamp, the combination of a pc-NIR LED and a warm white LED is the most appropriate to compensate for the missing waveband.

As shown in Figure 2a,b, a light source module was designed and manufactured with one warm white LED and 24 pc-NIR LEDs (SFH4776) for effectively reducing the discontinuity of the spectral response across the visible and NIR wavelengths. Since the maximum number of pc-NIR LEDs that can be installed in the same area as the light irradiation area of the TH-lamp was 24, a pc-NIR LED light source was developed by integrating 24 pc-NIR LEDs to maximize the output of the light source. The LED module has the layout of five series of five LEDs, five pairs of terminals for power connection, and the parabolic reflector to concentrate the radiation at the front of the LED panel, as depicted in Figure 2c. At the backend, the blower fan is mounted for the heat dissipation to reduce radiation instability. While emitting light, the five power terminals of the LED module were connected to the benchtop power supply, which was set up to provide the limited level of the forward current under the regulated voltage required for the stable operation of LED as shown in Figure 2d.

### 2.3. Data Acquisition System for VIS/NIR Spectroscopy

In order to capture the spectral intensities of the transmittance through an apple, an acquisition system was configured with a laptop computer, spectrometer (USB4000-VIS-NIR, Ocean Optics, Orlando, FL, USA), the fixture for transmittance measurement, two types of light sources (i.e., the developed and TH lamp) as shown in Figure 2d,e. In terms of optical layout, the light source was located at the equatorial level of an apple, where the VIS/NIR radiation passes through it. At the underneath of the apple, the measurement fixture was placed with an optical probe with a collimator lens, which is connected to an optical fiber. In terms of its optical pathway, the transmitted light from the left side of an apple is measured at the optical probe in the right side, which is connected to USB4000-VIS-NIR spectrometer with an optical slit width of approximately 200 µm.

### 2.4. Data Preparation for Spectra and SSC Measurement

A total of 160 Fuji apple samples were used, and the four equatorial positions of each apple were exposed to both light sources. For each light source, the measurements were performed 640 times, and the total 1280 spectra from the TH lamp and developed LED module were used for spectral analysis to evaluate the spectral characteristics. During the acquisition of spectra, both power supplements were maintained as similarly as possible, i.e., 20 W@12 V power for the TH lamp and 22 W@15 V for the VIS + NIR LED module.

In terms of the spectral resolution, the available wavelength range was a 471 to 1160 nm range, and the sampling step of the spectrograph was approximately 0.2 nm. For reference SSC measurement, the apple juice was measured using a digital refractometer (PR-101; Palette Series, ATAGO Co. Ltd., Tokyo, Japan). In summary, 640 spectra for each light source were acquired for calibration of the SSC of Fuji apples through PLS regression. Corresponding to the spectra of the apples, 640 SSC were measured from the juice from a slice of apple in one of fourth.

### 2.5. Algorithm of VIS/NIR Spectral Analysis

**Figure 3:**
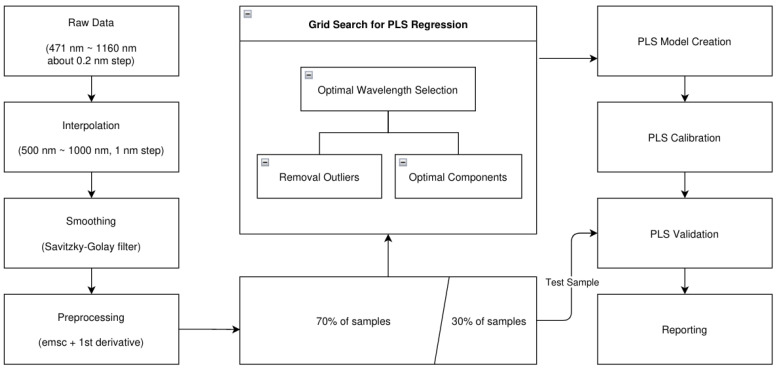
Algorithm of PLS regression comprising signal preprocessing (interpolation, smoothing, and preprocessing), sample splitting, grid search for optimal parameters, PLS calibration, validation, and reporting.

The optical characteristic of the TH lamp and the VIS + NIR LED module can be featured out through the spectral regression, which is calibrated and validated using the spectral transmittance and SSC from fruit. In the case of a Fuji apple providing the spectral intensities of the transmittance, and the SSC, a PLS regression was performed to predict SSC as shown in Figure 3. This process includes interpolation, smoothing, preprocessing, sample splitting, grid search for optimal parameters, PLS calibration, validation, and reporting.

#### 2.5.1. Signal Preprocessing

The data preparation of spectral data includes interpolation, smoothing, and preprocessing. The interpolation converts raw spectra with irregular wavelength steps into the uniformed spectra with 1-nm intervals, which is performed through signal filtering with a Gaussian filter having a standard deviation of 1 nm, and a spectral range of 471 to 1160 nm is regularized to 500–1000 nm. During the smoothing process, the interpolated spectra are reformed using the method described by Savitzky and Golay (1964) [26]. In preprocessing, the scattering effects are reduced by Extended Multiplicative Signal Correction (EMSC) followed by second derivates. As a result, the spectral interferences are eliminated.

#### 2.5.2. Data Splitting

The preprocessed spectral data and SSC measurements were separated into 70% and 30% for PLS calibration and validation, respectively; 70% of the samples were used to create the PLS model, and the remaining samples were used for validation.

Before the PLS calibration process, it is necessary to determine several parameters such as the number of components of the PLS model, the optimal wavelengths, and the removable outliers. For optimization of the PLS model, they must be determined by the position of these three parameters with minimum MSE. Their determination process involves a grid search to generate MSE logs along with three parameters. The grid search accumulates the MSE of PLC calibration with unseen validation data, and provides the optimal parameters, the component number of PLS, the optimal wavelengths, and removeable outliers, for the PLS calibration.

#### 2.5.3. PLS Validation and Reporting

With the samples (30% of the total samples) not used for PLS calibration, the PLS model was evaluated based on the correlation coefficient (R) and the mean square error of prediction (MSEP). For both calibration and validation, the performance of the PLS model was compared with respect to R, the MSEP, mean square error of calibration (MSEC), standard deviation of error for predication (SEP), standard deviation of error for calibration (SEC), ratio of the standard error of prediction to standard deviation (RPD), and the error bias.

## 3. Results

In order to determine the best candidate for replacement of a TH lamp among the available commercial pc-NIR LEDs such as SFH4735, SFH4736, SFH4776, and L1IG-0750100000000, it is reasonable to compare the spectral intensity and distribution of their radiation. Through the spectral comparison, SFH4776, which had a flatter and higher spectral intensity, was a better replacement for the TH lamp in the 650–1000 nm waveband.

For providing the full spectral coverage of the TH lamp, two types of LEDs were used to build the Vis + NIR LED module which can provide the radiation from 400 to 1000 nm; SFH4776 for near-infrared emission (650–1000 nm) and GW CSSRM2.EM (OSRAM GmbH, Munich, Germany), a warm-white LED, for the visible waveband (400–650 nm). For the evaluation of the feasibility of the LED module, spectral measurements were performed to acquire the transmittance via Fuji apples against a 20-W TH lamp, and then, PLS regression was performed for the estimation of SSC.

### 3.1. pc-NIR LED Comparison

**Figure 4:**
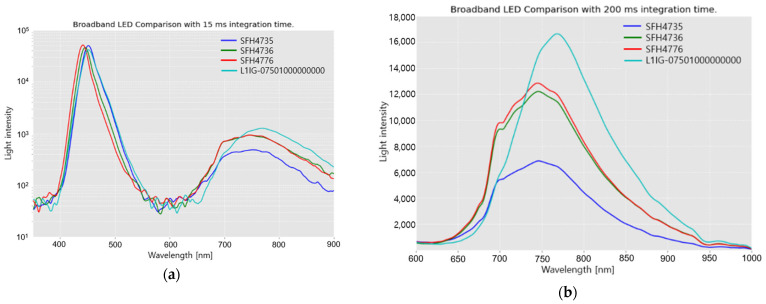
Spectral intensities of the radiation of pc-NIR LEDs (SFH4735, SFH4736, SFH4776, and L1IG-0750100000000): (**a**) Overall spectral intensity of radiation in the 400–900 nm range; (**b**) Enlarged image of the spectral intensity of radiation in the 600–1000 nm range.

As shown in Figure 4a, a pc-NIR LED emits radiation in a broad waveband of 400 to 1000 nm. All the spectra commonly exhibit two separate Gaussian peaks, which have a Gaussian-like graph with a peak at 450 nm originating from the excitation spectrum generated by the violet LED on the silicon chip. The emission spectrum in the range 600–900 nm is generated by phosphor, which is supposed to generate near-infrared fluorescence on excitation by the 450 nm peak radiation of the violet LED. Phosphor is deposited on the glass above the silicon chip of the pc-NIR LED through an evaporation process. In the fluorescence process within all the pc-NIR LEDs, the portion of the excitation by the violet LED is superior to the fluorescence emission through phosphor transformation. Hence, they still have considerable room for increasing the NIR emission by improving the density of phosphor, and the conversion efficiency.

In the 450-nm peak region, there is no significant difference in the intensity. Figure 4b presents a closer look at the fluorescence emission ranging from 600 to 1000 nm. In terms of the radiation intensity, L1IG-0750100000000 exhibits the highest peak at approximately 760 nm, whereas the others (SFH4735, SFH4736, and SFH4776) show relatively flattened spectra and lower peaks.

LEDs with flat and high-intensity spectra are a good replacement for the TH lamp as a light source for VIS/NIR spectroscopy. Furthermore, in most VIS/NIR instruments, the spectrometer includes a detector composed of CCD/CMOS devices, which shows an available spectral response between 400 and 900 nm. This is important for emission in the 600–700 nm region, rather than at 900 nm. Therefore, SFH4776 would be a better replacement for the TH lamp because of its flat and high intensity in the 600–700 nm region.

### 3.2. TH Lamp, and VIS + NIR LED Module Transmittance Comparison

As a part of the VIS + NIR LED module, SFH4776 was used for emitting NIR radiation (650–1000 nm), and the remaining visible region was covered by a warm white LED, GW CSSRM2.EM. The spectral radiation of the LED module with two types of LEDs, a warm white LED and 24 pc-NIR LED (SFH4776), is likely to be that of the TH amp.

**Figure 5:**
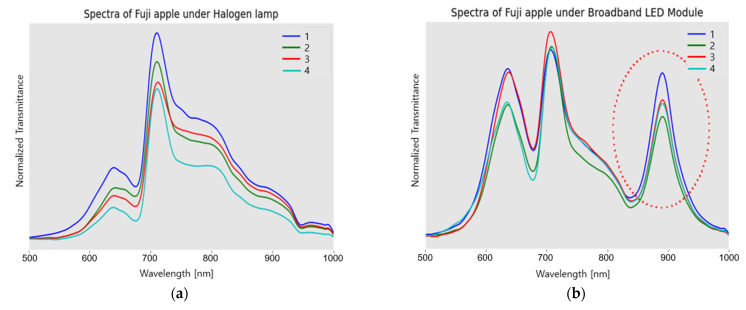
Spectral transmittance through a Fuji apple using the TH-lamp, and the Vis + NIR LED module: Spectra transmitted by radiation of (**a**) the TH lamp at the four equatorial positions of a Fuji apple; (**b**) the Vis + NIR LED module, including the second order diffraction of 450-nm peaked radiation from a blue LED device of SFH4776 and GW CSSRM2.EM; Numbers of 1–4 for the positional acquisition of one Fuji apple.

To compare the spectral characteristics, the spectra transmitted at the four equatorial locations of a Fuji apple were acquired, where they were illuminated by the TH lamp and VIS + NIR LED module, respectively. Figure 5a shows the typical transmittance spectra of Fuji apples generated by light penetration from a 20-W TH lamp, whereas Figure 5b shows the spectra generated by the VIS-NIR radiation from the VIS + NIR LED module. The two types of spectra commonly exhibit two main peaks at 650 nm and 720 nm, which reveal features imprinted by apple tissue [23,24,25]. However, even if the number of pc-NIR LEDs is higher than the warm white LED in the VIS + NIR LED module, the peak around 720 nm is not much higher than that at 650 nm in the comparison of the spectra using the TH lamp. It should also be noted that the 900-nm peak, enclosed by the dotted circle in Figure 5b, might be due to the second order diffraction of the 450-nm radiation from the blue LED device of SFH4776, and GW CSSRM2.EM. It was confirmed by the manufacturer that the second diffraction was caused by the grating design of the USB4000.

### 3.3. SSC Prediction Performance Using TH-Lamp and VIS + NIR LED Module

In order to feature out the spectroscopic effect of the light source, it is reasonable to investigate the estimation performance of the SSC of Fuji apples using the spectra. Therefore, this section describes the spectral analysis for the VIS + NIR LED module and TH-lamp. Two PLS models were created using the spectra of the Fuji apples generated by the radiation from a TH lamp and VIS + NIR LED module, respectively, and the SSC values measured from the juice of Fuji apples. Both were then compared with respect to R, the MSE, SPC, RPD, and bias. In total, 640 transmittance spectra for each of the VIS + NIR LED module and TH lamp were acquired from 160 Fuji apples, and 640 SSC values were used to build the PLS regression model for predicting the SSC; the four spectra obtained at the four equatorial positions correspond to the SSC value from a slice in a quarter of an apple.

**Figure 6:**
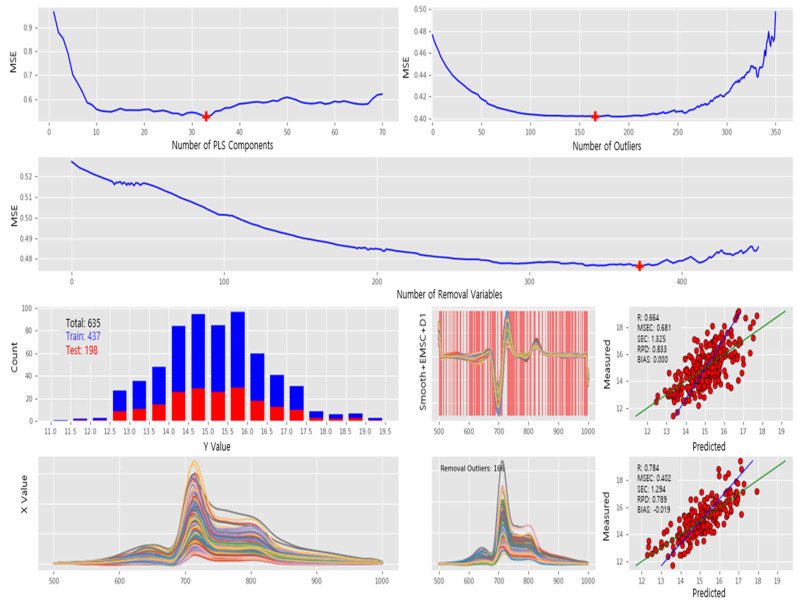
Preprocessing, data splitting, calibration, and validation of PLS model for spectra captured under TH lamp: (1st and 2nd rows) MSE plots along with the number of PLS components, removal outliers, and removal wavelengths as *x*-axis in clockwise direction. (3rd row) Sample distribution for PLS calibration and validation, preprocessed spectral intensity, and PLS calibration report. (4th row) Raw spectral intensity, removed outliers during PLS calibration, and PLS validation report.

In Figure 6, the spectra captured under the TH lamp light source and the entire process of PLS modeling, which includes the determination of the optimal parameters, calibration and validation, are described; the raw spectra and preprocessed spectra are depicted in the bottom row, respectively. In the middle, 635 spectra are split into a 7:3 ratio for PLS calibration and validation; both are equally distributed over the SSC values, and they are 437 and 198 spectra, respectively. In the top row of Figure 6, MSE fluctuation during PLC calibration is depicted as three MSE plots along with the number of the PLS components, removable wavelengths, and removable outliers as the *x*-axis. At this point, the optimal parameters for PLS calibration are determined as the number of PLS components (33), the number of removal wavelengths (372), and the number of removed outliers (166). In summary, the PLS model was calibrated with the optimal parameters. During the PLS calibration, the minimum MSE was approximately 0.4.

**Figure 7:**
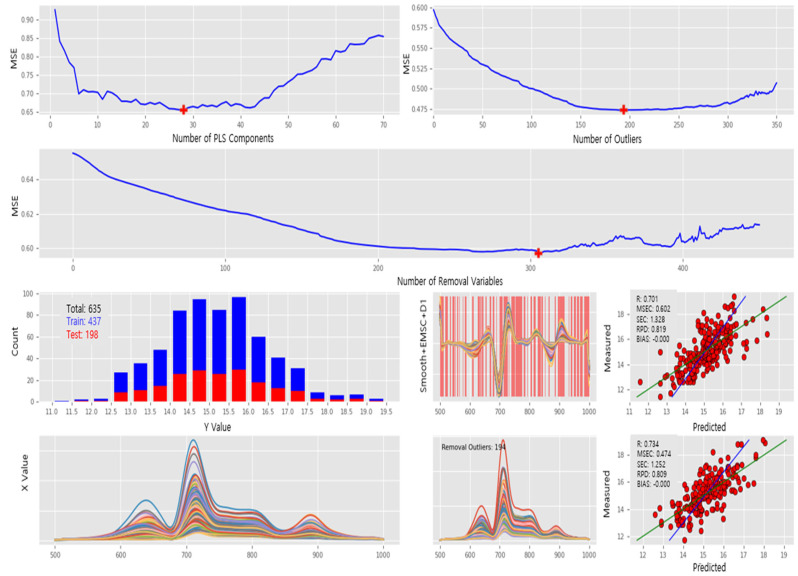
Preprocessing, data splitting, calibration, and validation of PLS model for spectra captured under VIS + NIR LED module: (1st and 2nd rows) MSE plots along with the number of PLS components, removal outliers, and removal wavelengths as *x*-axis in clockwise direction. (3rd row) Sample distribution for PLS calibration and validation, preprocessed spectral intensity, and PLS calibration report. (4th row) Raw spectral intensity, removed outliers during PLS calibration, and PLS validation report.

Similarly, for the spectra captured under the VIS + NIR LED module, PLS modeling was performed as shown in Figure 7. The spectral data were split into PLS calibration (437) and validation (198). For the PLS calibration, the following parameters were determined at the minimum MSE: the number of optimal PLS components (28), the number of removal wavelengths (305), and the number of removed outliers (194). Finally, the PLS model was created using the optimal parameters, and the corresponding MSE was approximately 0.47.

The PLS calibration and validation results for the TH lamp and the Vis + NIR LED module are summarized in Table 1. For the TH lamp, the PLS model, which was created with 33 PLS components, 129 wavelengths, 271 calibration spectra, was evaluated with 437 calibration and 198 validation spectra, respectively. For the Vis + NIR LED module, the PLS model with 28 PLS components, 196 wavelengths, and 243 calibration spectra was evaluated with 437 calibration and 198 validation spectra. The results demonstrate that the VIS + NIR LED module has great potential as a replacement for the TH lamp in miniature spectrometers and portable hand-held spectrometers.

## 4. Discussion

For both the TH lamp and VIS + NIR LED module, the spectra show commonly the spectral tendency of transmittance via the apple except for second order diffraction by 450 nm excitation. The R value of PLS calibration is lower than that of PLS validation because of MSE calculation for all calibration spectra even if the outliers are excluded during PLS calibration. In the same manner, the MSE of calibration is higher. In the comparison of R and MSE, the PLS model for the TH lamp does not show any significant performance and has rather a lower one than that of the VIS + NIR LED module; in the average of calibration and validation, the R values are 0.724 and 0.717, and the MSE are 0.541 and 0.538 for the TH lamp and VIS + NIR LED module, respectively. Thus, this study has the experimental limitation of the second-order diffraction; however, the developed VIS + NIR LED module seems to be replaceable for the TH lamp in the NIR spectral application ranging from 400 nm and 1000 nm.

## 5. Conclusions

In terms of the performance of the PLS regression, there are no remarkable differences in R and the MSE model between the TH lamp, and the VIS + NIR LED module; in the case of the R value, the TH lamp is not superior, but has a higher MSE than the developed one. The results of this study indicate the immense potential of the VIS + NIR LED as a replacement for the TH lamp in VIS-NIR spectroscopy, which can lead to the emergence of portable spectrometers and hyperspectral imaging devices.

## Figures and Tables

**Table 1:** R, MSE, Std. of Error, RPD, and Bias for PLS calibration, and validation of Fuji apple SSC prediction under TH lamp, and the VIS + NIR LED module.

	TH Lamp		VIS + NIR LED Module	
	Calibration	Validation	Calibration	Validation
R	0.664	0.784	0.701	0.734
MSE	0.681	0.402	0.602	0.474
Std. of Errors	1.325	1.294	1.328	1.252
RPD	0.833	0.789	0.819	0.809
Bias of Errors	0	−0.019	0	0

## Data Availability

Not applicable.
